# Comparing full immunisation status of children (0–23 months) between slums of Kampala City and the rural setting of Iganga District in Uganda: a cross-sectional study

**DOI:** 10.1186/s12913-023-09875-w

**Published:** 2023-08-14

**Authors:** Awa Jammeh, Michael Muhoozi, Asli Kulane, Dan Kajungu

**Affiliations:** 1 Department of Global Public Health, Karolinska Institutet, Stockholm, Sweden; 2 Edward Francis Small Teaching Hospital, Banjul, The Gambia; 3 Center for Health and Population Research, Makerere University, Kampala, Uganda; 4 Division of Epidemiology and Biostatistics, Department of Global Health, Faculty of Medicine and Health Sciences, Stellenbosch University, Stellenbosch, South Africa

**Keywords:** Full immunisation status, Slums, Rural area, Individual dose vaccines

## Abstract

**Background:**

Immunisation remains the most cost-effective public health intervention in preventing morbidity and mortality due to Vaccine-Preventable Diseases (VPDs). The study aims to compare the differences in immunisation coverage amongst children aged 0 to 23 months living in slums of Kampala city and Iganga as rural districts in Uganda.

**Methods:**

This study utilises data from a cross-sectional survey done in 2019 in the slums of Kampala City and the rural district of Iganga within the Health and Demographic Surveillance Site (HDSS). It included 1016 children aged 0–23 months and their parents. A logistic regression model was used to analyse the relationship between multiple independent variables and the binary dependent variables (fully immunised) using Stata statistical software. The measures of association were odds ratios reported with a corresponding 95% confidence interval.

**Results:**

Out of the 1016 participants, 544 participants live in the rural area and 472 participants in the slums. Slums had 48.9% (n = 231) of fully immunised children whilst rural areas had 43.20% (n = 235). The multivariate analysis showed that children living in slums are more likely to be fully immunised as compared to their counterparts in rural areas (Odds ratio:1.456; p = 0.033; CI:1.030–2.058). Immunisation coverage for BCG (98.9%), Polio 0 (88.2%), Penta1 (92.7%), and Pneumo1 (89.8%) were high in both settlements. However, the dropout rate for subsequent vaccines was high 17%, 20% and 41% for Penta, pneumococcal and rota vaccines respectively. There was poor uptake of the new vaccines with slums having 73.4% and 47.9% coverage for pneumococcal and rota vaccines respectively and rural areas had 72.1% and 7.5% for pneumococcal and rota vaccines respectively.

**Conclusion:**

The low full immunisation status in this study was attributed to the child’s residence and the occupation of the parents. Lack of education and poor access to messages on immunisation (inadequate access to mass media) are other contributing factors. Educational messages on the importance of immunisation targeting these underserved populations will improve full immunisation coverage.

**Supplementary Information:**

The online version contains supplementary material available at 10.1186/s12913-023-09875-w.

## Background

Immunisation remains the most cost-effective intervention in public health. Globally, vaccination currently prevents 2–3 million deaths annually as more than a billion children were vaccinated in the past decade [1]. There has been a significant reduction in childhood mortalities related to vaccine-preventable diseases (VPDs) from 5.1 million in 1990 to 1.8 million in 2017 [2]. The World Health Organisation (WHO) defines immunisation as the process whereby a person is made immune or resistant to infection, typically by the administration of a vaccine [3] through a process of giving antigenic material. Vaccination coverage on the other hand is defined by the WHO as “the proportion of a given population that has been vaccinated in a given period. It accounts for each vaccine and, for multi-dose vaccines, for each dose received” [4].

In May 2012, the World Health Assembly developed the Global Vaccine Action Plan (GVAP) to help avert millions of childhood mortality attributed to VPDs through equal access to vaccines by 2020 and beyond to tackle inequalities associated with access to life-saving vaccines, especially in Low and Middle Income Countries (LMICs) [5, 6]. Despite the WHO efforts, the coverage is still below the global target [1, 2, 7]. Studies on immunisation coverage in Africa have shown improved but suboptimal coverage in most settings [8–10]. Therefore, most countries are not in line with reaching the SDG target 3.2 of reducing childhood mortality [11].

Uganda has also succeeded in reducing child mortality, however, the level is still high at 40.564 deaths per 1000 live births as compared to the global target as of 2022 [12]. Most of the under-5 deaths are due to VPDs making it a public health concern. In 1983, the Ugandan National Expanded Program on Immunisation (UNEPI) was established in partnership with Global Alliance for Vaccines and Immunisation (GAVI) to achieve vaccination goals; following the vaccination schedule on S1 Table 1 [13]. The immunisation services are offered by trained healthcare workers in health facilities and through selected community outreaches. Periodical supplementary immunisation is carried out during outbreaks as well. The immunisation services aim to completely immunise children and women of childbearing age against Diphtheria, Hepatitis B infection, Polio, Whooping cough, Tuberculosis, Tetanus, Measles, Haemophilus influenza and Pneumococcal Infections whilst 10-year-old females and women of reproductive age are immunised against Human Papilloma Virus that causes Cancer of the Cervix and Tetanus [14] (S1 Table 1).

**Fig. 1:**
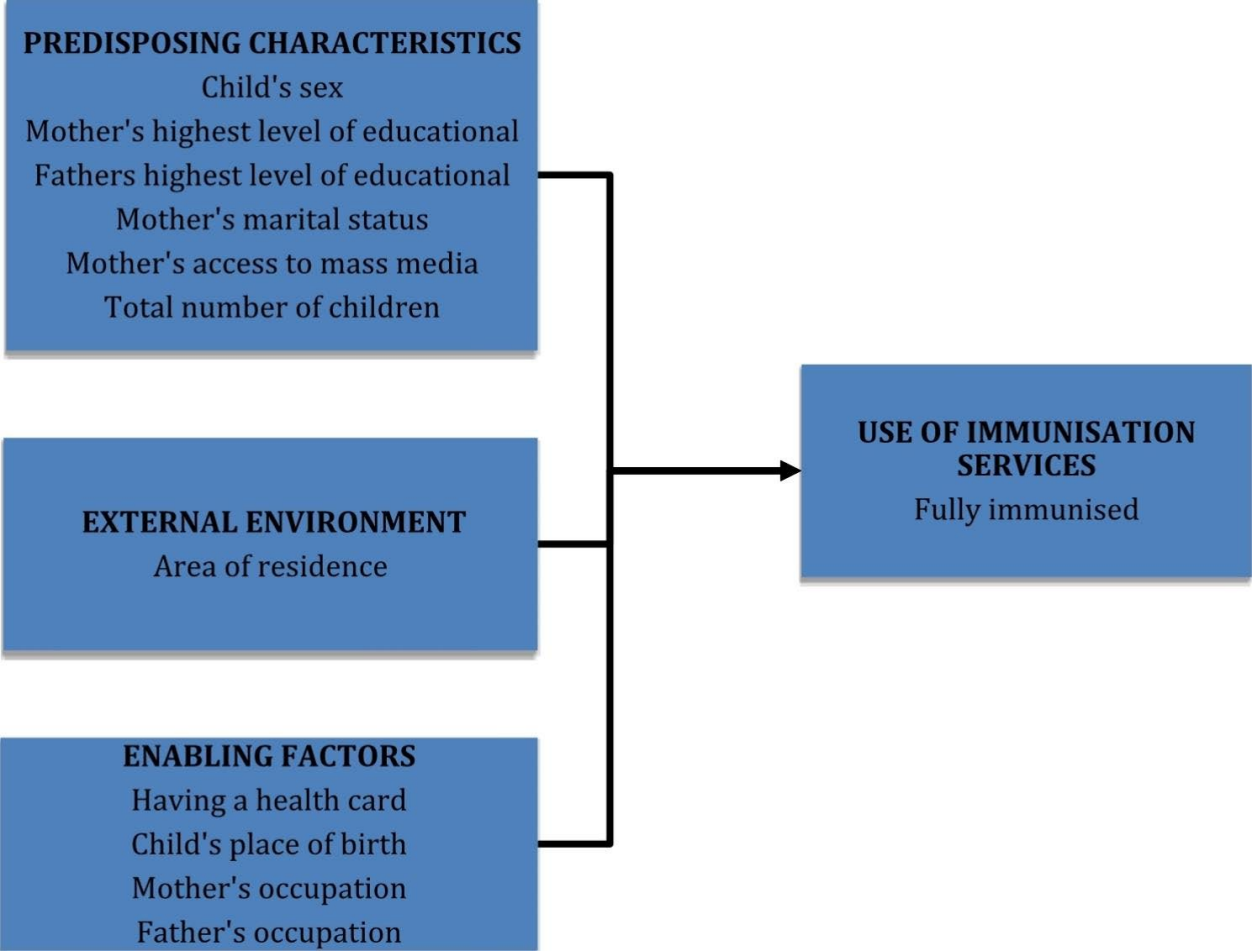
Modified Anderson’s behavioural model for healthcare utilisation. The variables are classified as predisposing characteristics, external environment and enabling factors. It shows how these factors influence the utilisation of health services (i.e. to be fully immunised)

Despite immense efforts by the government of Uganda and its developmental partners to increase immunisation coverage, a decline in immunisation coverage has been noticed. A 90% vaccine coverage is the national acceptable target however, only BCG (96%) is at the national acceptable level whilst the other vaccines are below the accepted levels [15] (S2 Fig. 1).

According to the WHO data on childhood immunisation coverage in Uganda, there has been a further decline in immunisation coverage in the past years and it has significantly dropped from 90% to below 80% as of 2022.

The full immunisation status of children cannot be assessed without discussing the factors that determine immunisation. As modernization grows with increasing urbanisation, more people tend to live in urban areas with many being marginalised in the slums and unable to access immunisation services. This is coupled with challenges in reaching remote rural populations with difficulty in technological development for the cold chain into the bargain [16].

Most studies have shown that the educational level of the parents, awareness of the availability of immunisation services, health-seeking behaviour, wealth index, place of residence, parents’ educational level and occupation and distance to the service delivery points are the main factors contributing to low coverage of full immunisation in children. In Sub-Saharan African settings, these individuals and contextual factors play an important role in improving the immunisation status of children.

Studies in Sub-Saharan Africa comparing coverage of full immunisation status of children in urban cities, slums and rural settlements showed that slums have better coverage compared to rural settings [17, 18]. Other studies showed significantly poor coverage of full childhood immunisation status in both slums and rural areas [19–21]. Many works of literature, however, studied coverage of full immunisation status in urban settings comparing it to the rural area with urban areas having better coverage [10, 22–28]. Literature has also shown that children from educated parents [10, 23, 28–30], parents with access to information from mass media [9, 31], and parents who are employed coupled [32, 33] with high wealth index [34] had higher chances of being fully immunised as compared to their counterparts.

Therefore, this study compares the differences in full immunisation status among children aged 0 to 23 months living in slums of Kampala city and Iganga District as rural districts in Uganda. It also highlighted some of the factors that influence the completion of all immunisation doses in both settings.

## Methodology

### Study design

The study utilised a comparative cross-sectional study between the slums of Kampala city and the Iganga District as the rural setting in Uganda. Unlike a longitudinal study, the data was collected at one point in time. With this type of study, we cannot prove causality [35].

### Study setting

Kampala is the capital city of Uganda and it is the largest city in the country with a population of about 1.5 million in the 2014 census [36]. According to Uganda Demographic and Health Survey UDHS data 2016, Kampala has childhood vaccination coverage of 51% with a high under-5 mortality of 64 deaths per 1000 live births [37].

Similarly, Iganga is one of the districts with many rural settings and has a population of over 500,000 people as of the 2014 census [38]. About 38% of people aged 18 and above are illiterate with a higher number being females; and 20% of the people are 5 km away from the nearest public health facility [38]. Generally, under-5 mortality in rural Uganda is at 68 deaths per 1000 live births in 2016 which is higher than the urban under-5 mortality.

The slums in this study are in Kampala city. Rapid urbanisation has led to increased slums in many urban settlements. These slums are usually marginalised and lack access to proper health care services. This could be the cause of high childhood mortality in Kampala city [39]. Overall, childhood mortality in Uganda has decreased over the past years. It has declined to 40.564 deaths per 1000 live births in 2022. However, these are far apart from the global targets of meeting SDG 3.2 which aspires to reduce avoidable death in newborns and children under the age of five.

### Data source

This study relied on a cross-sectional survey done in 2019 in the slums of Kampala city and the rural settings of Iganga District within the Iganga Mayuge Health and Demographic Surveillance Site (HDSS) catchment area. Data collection lasted for about three [3] months. The survey employed a simple random sampling of villages and households with eligible children. Selected households were interviewed to obtain data on the child and their parents. Immunisation status data for a child was taken from the welfare card record and for children without the cards; verbal reports from the mother were used.

The Iganga Mayuge Health and Demographic Surveillance System (IMHDSS) operates within the Iganga and Mayuge District in Eastern Uganda covering seven sub-counties and 65 villages. The IMHDSS collects bi-annual data on basic demographic events (births, migration, marriage and deaths) of all individuals in the demographic surveillance area [40].

#### Dependent variable

The child’s immunisation status was the dependent variable, and the information was either taken from the child’s welfare card or the mothers’ recall if the card was not available. The dependent variable had a binary outcome as to whether the child is fully immunised or not. A child was regarded as fully immunised when he/she received all the basic vaccines according to age as required by the national schedule. Any child that misses a dose of the vaccine according to age was regarded as not fully immunised. Children without vaccination cards and whose mothers could not remember their vaccination status were also regarded as not immunised.

#### Independent variables

The area of residence (urban or rural) was the explanatory variable of interest and a stratifying variable.

The **predisposing characteristics** include the child’s sex, mother’s marital status, parent’s highest level of education attained, mother’s access to mass media and number of children in the family. In the analysis, the observations for the child’s sex were male or female. Marital status was represented as 1 = in union, 2 = not in union. The mother’s access to media information was also categorised and analysed as to whether the mother heard information about immunisation campaigns and whether she also heard information about newly introduced vaccines (pneumococcal and rota vaccine).

Parents’ highest educational level attained includes both mother’s and father’s educational level. The observations include none (no education), primary, secondary, and tertiary. Finally, the **enabling factors** included the child’s place of birth, the child having a health card and parents’ occupation. The child’s place of birth was either in the hospital or outside the hospital. The parents’ occupation includes both the mother and father and was represented as 1 = small home business, 2 = market vendors, 3 = professional, 4 = other (farmer, unemployed etc.). For health card, it includes children with health card, children without but has mother’s recall and children without health card and no maternal recall.

A conceptual model first developed in the 1960s by Ronald M. Andersen was adopted in this study. This model aims at denoting the factors that lead to an individual’s use of health services which is divided into three characteristics. The characteristics include predisposing factors, enabling factors and need factors [41]. This model has been widely used in assessing the determining factors of health service utilisation (immunisation coverage as well) [27, 42, 43]. Therefore, this study modified Anderson’s model to characterise its variable. Area of residence (rural/urban) was the explanatory variable of interest in this study as it was comparing the immunisation status of two residential settings. However, covariates were classified and assessed as illustrated in the framework below (Fig. 1).

### Data management and statistical analysis

Data was electronically collected on the Open Data Kit (ODK) platform. Preliminary data management including cleaning, manipulations and edits were done in Microsoft Excel and statistical analysis was done using Stata version 16.1 software. Descriptive statistics were produced to summarise categorical variables that were presented as frequencies and percentages while continuous variables were presented as means and standard deviations. For coverage of individual vaccines, proportions with their 95% confidence intervals were used to report vaccination coverage in both settlements.

A logistic regression model was fitted to analyse the relationship between multiple independent variables and the binary dependent variables (fully immunised). A child was classified as fully immunized if they received all the recommended vaccines at an appropriate age. A binomial distribution was adopted for the two possible outcomes either “fully immunised” (success) or “not fully immunised” (failure). Univariate logistic regression was conducted to establish the relationship between full immunisation status and each independent variable. Furthermore, multivariate logistic regression was also conducted to know the effect of the independent variables on the full immunisation coverage. The measures of association in this analysis were odds ratios reported with a corresponding 95% confidence interval and the p-value were used to determine the precision of the estimate and the statistical significance.

## Results

### Descriptive characteristics of the participants

The total population of 1016 participants were studied and out of which, 544 participants live in the rural Iganga District and 472 participants are from the urban slums of Kampala city. The percentage of fully vaccinated children in slums and that in rural district presented 53% of the children that participated in the study as shown in S3 Fig. 2. Slums had 48.9% of fully immunised children whilst rural areas had 43.20% as shown in Table 1.

**Table 1:** Description of study population

Characteristics	Rural, n (%)	Urban Slum, n (%)
**Child received all vaccines recommended**		
No	309 (56.80)	241 (51.06)
Yes	235 (43.20)	231 (48.94)
**Health card for the child available**		
Yes, seen	388 (71.32)	289 (61.23)
Yes, not seen	150 (27.57)	150 (31.78)
No	6 (1.10)	33 (6.99)
**Where child was born**		
Hospital	371(68.20)	366(77.54)
Outside hospital	173(31.80)	106(22.46)
**Mother’s Occupation**		
Small home business	135(25.19)	110(24.39)
Market vendor	32(5.97)	25(5.54)
Professional	49(9.14)	22(4.88)
Other(specify)	320(59.70)	294(65.19)
**Father’s occupation**		
Small home business	54(10.25)	78(17.85)
Market vendor	54(10.25)	35(8.01)
Professional	143(27.13)	72(16.48)
Other	276(52.37)	252(57.67)
**Sex of the child**		
Female	257(47.24)	243(51.48)
Male	287(52.76)	229(48.52)
**Mother’s highest level of education**		
None	21(3.88)	31(6.68)
Primary	157(29.02)	142(30.60)
Secondary	300(55.45)	228(49.14)
Tertiary	63(11.65)	63(13.58)
**Father’s highest level of education**		
None	10(2.18)	21(5.48)
Primary	45(9.83)	70(18.28)
Secondary	233(50.87)	171(44.65)
Tertiary	170(37.12)	121(31.59)
**Marital status of child’s mother**		
Not in union	67(13.29)	116(25.44)
In union	437(86.88)	340(74.56)
**Respondent has heard messages about immunisation campaign messages**		
Yes	152(29.63)	135(29.16)
No	361(70.37)	328(70.84)
**Respondent has heard messages about new vaccines-pneumococcal and Rota**		
Yes	202(39.45)	23(4.98)
No	310(60.55)	439(95.02)

**Abbreviation**: **n**-number

As shown in Table 1, mothers in both settlements attained a secondary level of education of 55.5% and 49.14% in rural and slums respectively. A small percentage of the rural dwellers were not educated (3.9%) as well as those living in the slums (6.7%). Similarly, most fathers from both settlements attained a secondary level of education whilst only small percentages were not educated. A greater majority of the parents that participated were doing non-professional jobs (market vendor, small home business, farming etc.) in both settlements. A considerable number of mothers (87% and 65% from both rural and slums respectively) were in a union and living with their spouses. However, rural settlers had more women in a union than women not in a union compared to the slum dwellers. A higher percentage of parents in both communities had not heard about messages on immunisation campaigns and messages about the newly introduced vaccines (poor access to mass media).

A higher percentage of rural dwellers had health cards as compared to their slums counterparts of 71% and 61% respectively. However, hospital births were more in the slums than the rural settings.

### Comparing vaccination coverage in slums and rural settlements

The individual vaccine dose percentages and the vaccination dropout rate and the uptake of the new vaccines are shown in Table 2. Immunisation status for BCG, Polio0, Penta1, and Pneumo1 were high in both settlements. BCG had 97.9% coverage in slums and 99.5% coverage in rural areas. The slums had a higher coverage of 97.1% for Penta 1 and a lower coverage of 80.2% for Penta 3 indicating a dropout rate of 17%. Similarly, the rural area also had a dropout rate of 17% for the Pentavalent vaccine. In slums, pneumococcal vaccines had a coverage range of 93.3% and 73.4% for pneumo 1 and 3 respectively indicating a dropout rate of about 20%. The rural area had slightly lower percentages for pneumococcal vaccine. The percentages for Rota 1 were low and almost the same in both settlements. However, Rota 3 coverage was low in the slums (47.9%) and significantly lower in rural areas (7.5%). Generally, the Rota vaccine had a high dropout rate of 41%. The percentages for the measles vaccine were 53.8% and 44.4% for the slums and rural areas respectively.

**Table 2:** Coverage of individual vaccine doses comparing Urban slums and Rural area

	Area of Residence		
Individual doses	Urban Slums coverage (%)	Rural coverage (%)	Overall, % (95% Confidence Interval)
BCG	97.9	99.5	98.9 (97.6–99.5)
Polio 0	87.5	90.1	88.2 (85.2–90.6)
Penta 1	97.1	91.4	92.7 (90.3–94.6)
Pneumo 1	93.9	89.6	89.8 (87.0–92.1)
Rota 1	71.5	70.8	67.6 (63.5–71.4)
Polio 3	78.7	70.9	72.1 (68.2–75.7)
Penta 3	80.2	74.3	74.6 (70.8–78.1)
Pneumo 3	73.4	72.1	70.1 (66.2–73.8)
Rota 3	47.9	7.5	26.4 (22.9–30.3)
Measles	53.8	44.4	48.7 (44.8–52.9)

**Abbreviations: BCG** – Bacillus of Calmette and Guerin; **Penta** – Pentavalent vaccine (Diphtheria, Pertussis, Tetanus, Haemophilus Influenza B, Hepatitis B); **Pneumo** – Pneumococcal Conjugate Vaccine (PCV); **Rota** – Rotavirus

The logistic regression was fitted to assess the association between the dependent variable (Full immunisation according to age) with the selected independent variable. The results interpreted are only for the multivariate model with no interaction terms as shown in Table 3. Children living in slums were 45% more likely to be fully immunised compared to their counterparts in rural settings (aOR = 1.456; 95% CI:1.030–2.058, p-value = **0.033**) and male children had a 21% higher chance of being fully immunised compared to females (aOR = 1.208; 95% CI: 0.889–1.641, p-value = 0.227) but this was not statistically significant. Although not significant, children whose mothers were more educated had lower chances of being fully immunised compared to those whose mothers had no education at all. A similar pattern is observed for fathers’ education status. Mothers in any marital union were 31% less likely to have fully immunised Children compared to those not in a marital union (aOR = 0.692; 95% CI: 0.455–1.053, p-value = 0.086) but was not statistically significant.

**Table 3:** Univariate and multivariate logistic regression model for full immunisation with selected determinants

Full Immunisation	OR (95% CI)	p-value	aOR(95% CI)	p-value
**EXTERNAL FACTORS**				
**Area of residence**				
Rural	Reference		Reference	
Urban Slum	1.260 (0.984–1.615)	**0.048**	1.456 (1.030–2.058)	**0.033**
**PREDISPOSING FACTORS**				
**Sex**				
Female	Reference		Reference	
Male	1.317 (1.028–1.687)	**0.029**	1.208 (0.889–1.641)	0.227
**Mother’s educational level**				
None	Reference		Reference	
Primary	0.796 (0.441–1.437)	0.450	1.179 (0.540–2.571)	0.680
Secondary	0.682 (0.385–1.208)	0.190	0.881 (0.409–1.911)	0.754
Tertiary	0.602 (0.314–1.154)	0.127	0.747 (0.310–1.803)	0.517
**Father’s educational level**				
None	Reference		Reference	
Primary	0.704 (0.317–1.562)	0.388	0.840 (0.332–2.128)	0.713
Secondary	0.710 (0.340–1.478)	0.360	0.971 (0.407–2.321)	0.948
Tertiary	0.665 (0.316-1.400)	0.283	0.960 (0.387–2.381)	0.929
**Mother’s marital status**				
Not in union	Reference		Reference	
In union	0.743 (0.538–1.026)	0.071	0.692 (0.455–1.053)	0.086
**Heard about immunisation Campaigns**				
Yes	Reference		Reference	
No	1.257 (0.952–1.660)	0.107	1.224 (0.846–1.772)	0.283
**Heard about new vaccines**				
Yes	Reference		Reference	
No	0.950 (0.705–1.281)	0.737	0.829 (0.548–1.254)	0.375
**Total children**				
1–3	Reference		Reference	
4–7	1.223 (0.900-1.663)	0.199	1.388 (0.946–2.041)	0.096
8–12	0.772 (0.250–2.381)	0.652	0.553 (0.096–3.197)	0.508
**ENABLING FACTORS**				
**Health card**				
Yes, seen	Reference		Reference	
Yes, not seen	1.372 (1.045–1.803)	**0.023**	1.192 (0.851–1.672)	0.307
No	1.538 (0.805–2.940)	0.193	1.376 (0.569–3.325)	0.479
**Child’s place of birth**				
Hospital	Reference		Reference	
Outside hospital	1.062 (0.806-1.400)	0.669	0.991 (0.702–1.398)	0.957
**Mother’s occupation**				
Small home business	Reference		Reference	
Market vendor	2.661 (1.466–4.830)	**0.001**	1.976 (0.966–4.044)	0.062
Professional	1.348 (0.792–2.295)	0.272	1.592 (0.806–3.147)	0.181
Other	1.345 (0.994–1.818)	0.054	1.369 (0.942–1.988)	0.099
**Father’s occupation**				
Small home business	Reference		Reference	
Market vendor	1.524 (0.887–2.621)	0.127	1.716 (0.829–3.552)	0.146
Professional	1.296 (0.835–2.011)	0.247	1.858 (1.046–3.297)	**0.034**
Other	1.281 (0.869–1.887)	0.211	1.641 (0.997–2.702)	**0.051**

**Abbreviations: OR**- odds ratio, **aOR**-adjusted odds ratio

A caretaker hearing about immunisation campaigns did not increase the child’s chances of being fully immunized while the Children whose caretaker had heard about new vaccines were more likely to be fully vaccinated. Children coming from larger families (more than seven children in a household) were less likely to be fully immunized compared to those whose families were smaller (less than 4). Children who did not have a health card but had mother’s recall of immunisation during the interview had higher chances of being fully immunized compared to those who had cards. Children born outside the hospital had lower chances (10% less likely) of being fully immunized compared to those born in the hospital (aOR = 0.991, 95%CI: 0.702–1.398, p-value = 0.957).

## Discussions/analysis

This paper compared the state of immunisation coverage in the slums of Kampala the capital city of Uganda with coverage in the rural-based district of Iganga in Eastern Uganda for children aged of 0–23 months. Additionally, the study investigated how full immunisation status is influenced by a range of factors grouped as external environment, predisposing, and enabling in nature. Just over half of the children (53%) were fully immunised against vaccine-preventable diseases which was considerably low compared to the global target of 90% coverage [44] which could be responsible for high morbidity and mortality rates among infants and children.

### External environment (area of residence) influences a child’s full immunisation status

This study has shown that children living in slums had a higher chance of being fully immunised as compared to children in rural areas. This could be because slum dwellers had a higher percentage of hospital deliveries. Studies have shown that children delivered at a health facility usually have a full immunisation status [6]. A similar finding was made in Nigeria where it was established that slum dwellers had better coverage than rural settlers [17]. A recent study in India comparing urban and rural areas showed a significant difference with urban areas having a higher coverage. The study further compared the slums with the rural area and concurred that slum dwellers had better coverage despite their poor socioeconomic status [18].

The higher chance of full immunisation in slums compared to rural could also be due to the easier access to immunisation services in urban areas than the rural area as the rural settlements might be hard to reach. As discussed above, 20% of the rural settlers live more than 5 km from a health facility. It could also be because slum settlers had information on new vaccines. Therefore, they might have education on information on immunisation and its importance.

### Role of area of residence

Furthermore, full immunisation status for individual vaccines was also influenced by the child’s area of residence with children living in slums having a better coverage of the individual vaccines as compared to the rural dwellers. However, there was poor uptake of the 3rd doses of most vaccines in both settlements. This indicates a significant dropout rate of the vaccines. The study showed a huge gap (44% decline) in slum vaccine coverage between BCG birth dose vaccine (97,9%) and measles vaccine (53.8%) given at 9 months. A low uptake of measles vaccine at 9 months is a challenge in obtaining full immunisation coverage. The reason in this study could be attributed to inadequate access to mass media as most mothers have not heard about immunisation campaigns. Therefore, campaigns on vaccination need to be improved to improve parents’ consciousness of vaccine uptake. Similar results were seen in research done in the slums of Nairobi [19].

Likewise, a drop in coverage was noticed at 8.8%, 16.9%, 20.5% and 23.6% from the first to the 3rd dose of Polio vaccine, Penta, Pneumo and Rota vaccines respectively. The rural areas even had a higher dropout rate in the individual vaccines mentioned. A similar problem has been seen in rural Hoima District, Uganda showing a low coverage of measles as compared to the BCG birth dose vaccine and a dropout rate of 28.5% on DPT (between DPT 1 and 3) [21]. These high dropout rates and gaps indicate a high prevalence of missed opportunities in childhood vaccination which is a global public health concern and an obstacle to attaining the Sustainable Development Goal of reducing child mortality. The knowledge of parents on the importance of immunisation is the key driver in achieving full immunisation. The uptake of new vaccines in both settings was also assessed and there was significantly low coverage of Pneumococcal vaccine and Rota vaccine with worse uptake in the rural area. This could be due to the poor dissemination of messages on the availabilities of immunisation services in the communities and the importance of having children take them. Both slums and rural areas need promotions and activities geared towards educating mothers on the importance of having their children receive all the vaccines on time. The lack of sensitization which is shown as parents not having adequate knowledge of childhood immunisation might be an important factor in both poor uptake of new vaccines and the high dropout rate of the other vaccine.

One might expect slums to have a better full childhood immunisation coverage than the rural areas as it is in the urban area and we can argue that it should be closer to healthcare resources. However, the evidence presented in this study shows the contrary. The slum dwellers are marginalised and have little to no access to services. Due to the rapid urbanisation of Kampala city, many people searching for better livelihood tend to live in slums as it is more affordable and accessible for the poor and unemployed [45]. There is no plan in place to organise the needs of the slum dwellers. The lack of services and individual attitudes become contributing factors to poor full-childhood immunisation in slums as the purpose of living shifts to daily survival instead of providing the merits of responsible and organised living, planning, compliance and proper service delivery [46]. This can be a leading factor in hindering progress in achieving good health. Therefore both slums and rural areas have a very low uptake of childhood vaccines.

### Predisposing factors to attaining full immunisation status

Continuing with the predisposing factors, it showed that male children tend to be fully immunised compared to female children. The mother’s marital status was not statistically significant in this study. However, other studies have shown the contrary that married women tend to have their children fully immunised as well. This is because married women tend to have better healthcare-seeking behaviour [47, 48]. Moreover, the involvement of the partner could contribute to the financial support and the utilisation of healthcare.

### Enabling factors to attain full immunisation status

Finally, the enabling factors were mostly associated with full immunisation. Children without health cards had a higher chance of full immunisation. Children reported not having a health card but received immunisation could be because of a recall bias of the parents. The findings further suggest that parents’ educational attainment had no impact on full immunisation and that could be the reason why mothers that were market vendors had higher chances of immunising their children compared to those in a professional job. This could be because the market vendors are self-employed and can therefore have time to immunise their children.

### Limitations of this study

A limited set of variables were used to study the determinants of immunisation coverage. Factors such as mother’s antenatal visits could not be assessed. Maternal recall was used to determine whether a child was fully immunised or not and this increases the chances of recall bias in the study. Unlike a longitudinal study, the data was collected at one point in time. With this type of study, we cannot prove causality [35].

## Conclusion and recommendation

We have found out that the low full immunisation coverage in this study was mainly attributed to the residence of the child and the occupation of the parents. However, lack of education and poor access to messages on immunisation (inadequate access to mass media) are also contributing factors. Other factors that can be studied further could be related to poor supply chain, poor healthcare delivery system, and religious and cultural beliefs. Therefore, policies on improving the education of the population on the importance of immunisation need to target these populations to improve their immunisation coverage. Immunisation programs can be done through the improvement of primary health care facilities, training more staff and improving awareness by broadcasting on radios, television and announcing in markets/open places where people gather. These will improve access to information and thereby improve knowledge of the importance of immunisation which is an important factor in determining a child’s immunisation status [49]. Immunisation processes should also include integrating maternal and child health services to improve hospital deliveries, having health cards and immunising children.

### Electronic supplementary material

Below is the link to the electronic supplementary material.

Supplementary Material 1

## Data Availability

The data that support the findings of this study are available from Makerere University Center for Health and Population Research (MUCHAP) but restrictions apply to the availability of the data, which were used under the license for the current study and so are not publicly available. Data are however available from the authors upon reasonable request and with permission of MUCHAP.

## Abbreviations

aOR: Adjusted Odds Ratio
BCG: Bacillus Calmette-Guérin
GAVI: Global Alliance for Vaccines and Immunisation
GVAP: Global Vaccine Action Plan
HDSS: Health and Demographic Surveillance Site
IMHDSS: Iganga Mayuge Health and Demographic Surveillance System
LMICs: Low and Middle Income Countries
ODK: Open Data Kit
PENTA: Pentavalent Vaccine
PNEUMO: Pneumococcal Conjugated Vaccine
ROTA: Rotavirus Vaccine
SDGs: Sustainable Development Goals
UDHS: Uganda Demographic and Health Survey
UNEPI: Ugandan National Expanded Program on Immunisation
VPDs: Vaccine-Preventable Diseases
WHO: World Health Organisation

## References

[1] Immunization coverage [Internet]. [cited 2021 Feb 15]. Available from: https://www.who.int/en/news-room/fact-sheets/detail/immunization-coverage.

[2] Vanderslott S, Dadonaite B, Roser M, Vaccination - Our World in Data. Our World in Data [Internet]. 2013 May 10 [cited 2021 Feb 15]; Available from: https://ourworldindata.org/vaccination.

[3] World Health Organization. Immunization [Internet]. [cited 2021 Feb 15]. Available from: https://www.who.int/topics/immunization/about/en/.

[4] World Health Organization. Vaccination Coverage Cluster Surveys: Reference Manual. 2015 [cited 2021 May 12]; Available from: https://www.who.int/immunization/monitoring_surveillance/Vaccination_coverage_cluster_survey_with_annexes.pdf.

[5] World Health Organization. Global Vaccine Action Plan 2011–2020. WHO Library Cataloguing-in-Publication Data [Internet]. 2013 [cited 2021 Feb 17]; Available from: https://www.who.int/publications/i/item/global-vaccine-action-plan-2011-2020.

[6] Bustreo F, Okwo-Bele JM, Kamara L (2015). World Health Organization perspectives on the contribution of the Global Alliance for Vaccines and immunization on reducing child mortality. Arch Dis Child.

[7] Lim SS, Stein DB, Charrow A, Murray CJL (2008). Tracking progress towards universal childhood immunisation and the impact of global initiatives: a systematic analysis of three-dose diphtheria, tetanus, and pertussis immunisation coverage. Lancet.

[8] de Figueiredo A, Were F (2019). Local trends in immunisation coverage across Africa. Lancet.

[9] Tesema GA, Tessema ZT, Tamirat KS, Teshale AB (2020). Complete basic childhood vaccination and associated factors among children aged 12–23 months in East Africa: a multilevel analysis of recent demographic and health surveys. BMC Public Health.

[10] Bbaale E (2013). Factors influencing childhood immunization in Uganda. J Health Popul Nutr.

[11] Roser M, Ritchie H, Dadonaite B, Child and Infant Mortality-Our World in Data. Our World in Data [Internet]. 2013 May 10 [cited 2021 Feb 15]; Available from: https://ourworldindata.org/child-mortality#child-mortality-is-an-everyday-tragedy-of-enormous-scale-that-rarely-makes-the-headlines.

[12] Mortality rate, under-5 (per 1,000 live births) - Uganda |Data [Internet]. [cited 2021 Feb 18]. Available from: https://data.worldbank.org/indicator/SH.DYN.MORT?locations=UG.

[13] Uganda. |Gavi, the Vaccine Alliance [Internet]. [cited 2021 Feb 18]. Available from:https://www.gavi.org/programmes-impact/country-hub/africa/uganda.

[14] The Republic of Uganda Ministry of Health, UNICEF, World Health Organisation. The Role of Schools In Promoting Routine Immunisation. 2017 Oct [cited 2021 Feb 17]; Available from: https://www.unicef.org/uganda/media/2781/file/The%20role%20of%20schools%20in%20promoting%20routine%20immunization.pdf.

[15] The Republic of Uganda Ministry of Health, World Health Organisation, UNICEF. A Guide for National and District Leaders to Promote Routine Immunisationin Uganda. 2016 [cited 2021 Feb 18]; Available from: https://www.unicef.org/uganda/media/2816/file/A%20guide%20for%20national%20and%20district%20leaders%20to%20promote%20routine%20immunisation%20in%20Uganda.pdf.

[16] United Nations Children’s Fund (UNICEF). Immunization Roadmap 2018–2030. 2018 Sep [cited 2021 Feb 17]; Available from:https://www.unicef.org/sites/default/files/2019-01/UNICEF_Immunization_Roadmap_2018.pdf.

[17] Obanewa OA, Newell ML (2020). The role of place of residency in childhood immunisation coverage in Nigeria: analysis of data from three DHS rounds 2003–2013. BMC Public Health.

[18] Mukerji SVB (2017). Socio economic factors effecting immunisation coverage: focus areas. IJMEDPH.

[19] Mutua MK, Kimani-Murage E, Ettarh RR (2011). Childhood vaccination in informal urban settlements in Nairobi, Kenya: who gets vaccinated?. BMC Public Health.

[20] Kesarwani P, Singh N, Keshari SS, Dixit S (2017). Cross sectional study of immunization coverage in urban slum areas of Lucknow region. Int J Community Med Public Health.

[21] Malande OO, Munube D, Afaayo RN, Annet K, Bodo B, Bakainaga A (2019). Barriers to effective uptake and provision of immunization in a rural district in Uganda. PLoS ONE.

[22] Kagoné M, Yé M, Nébié E, Sie A, Schoeps A, Becher H (2017). Vaccination coverage and factors associated with adherence to the vaccination schedule in young children of a rural area in Burkina Faso. Glob Health Action.

[23] Adedokun ST, Uthman OA, Adekanmbi VT, Wiysonge CS (2017). Incomplete childhood immunization in Nigeria: a multilevel analysis of individual and contextual factors. BMC Public Health.

[24] Tesfaye TD, Temesgen WA, Kasa AS (2018). Vaccination coverage and associated factors among children aged 12–23 months in Northwest Ethiopia. Hum Vaccin Immunother.

[25] Etana B, Deressa W (2012). Factors associated with complete immunization coverage in children aged 12–23 months in Ambo Woreda, Central Ethiopia. BMC Public Health.

[26] Munthali AC (2007). Determinants of vaccination coverage in Malawi: evidence from the demographic and health surveys. Malawi Med J.

[27] Sarker AR, Akram R, Ali N, Chowdhury ZI, Sultana M. Coverage and Determinants of full immunization: Vaccination Coverage among Senegalese Children.Med (Kaunas).2019;55(8). 10.3390/medicina55080480 PMC6723170 31416213

[28] Nour TY, Farah AM, Ali OM, Osman MO, Aden MA, Abate KH (2020). Predictors of immunization coverage among 12–23 month old children in Ethiopia: systematic review and meta-analysis. BMC Public Health.

[29] Noh J-W, Kim Y-M, Akram N, Yoo K-B, Park J, Cheon J (2018). Factors affecting complete and timely childhood immunization coverage in Sindh, Pakistan; a secondary analysis of cross-sectional survey data. PLoS ONE.

[30] Anello P, Cestari L, Baldovin T, Simonato L, Frasca G, Caranci N (2017). Socioeconomic factors influencing childhood vaccination in two northern italian regions. Vaccine.

[31] Melovic B, Jaksic Stojanovic A, Vulic TB, Dudic B, Benova E. The impact of online media on parents’ Attitudes toward Vaccination of Children-Social Marketing and Public Health.Int J Environ Res Public Health.2020;17(16). 10.3390/ijerph17165816 PMC7459934 32796740

[32] Oleribe O, Kumar V, Awosika-Olumo A, Taylor-Robinson SD (2017). Individual and socioeconomic factors associated with childhood immunization coverage in Nigeria. Pan Afr Med J.

[33] Tamirat KS, Sisay MM (2019). Full immunization coverage and its associated factors among children aged 12–23 months in Ethiopia: further analysis from the 2016 Ethiopia demographic and health survey. BMC Public Health.

[34] Debie A, Amare G, Handebo S, Mekonnen ME, Tesema GA (2020). Individual- and community-level determinants for complete vaccination among children aged 12–23 months in Ethiopia: a Multilevel Analysis. Biomed Res Int.

[35] Levin KA (2006). Study design III: cross-sectional studies. Evid Based Dent.

[36] Uganda: Administrative Division (Regionsand Districts) - Population Statistics, Charts and Map [Internet]. [cited 2021 Feb 23]. Available from: https://www.citypopulation.de/en/uganda/admin/.

[37] Uganda Bureau of Statistics (UBOS)., ICF. Uganda Demographic and Health Survey 2016. 2018 [cited 2021 Feb 23]; Available from:http://www.ubos.org.

[38] Uganda Bureau of Statistics. National Population and Housing Census 2014 Area Specific Profiles. 2017 Apr [cited 2021 Feb 23]; Available from: http://www.ubos.org/.

[39] Kamya C, Namugaya F, Opio C, Katamba P, Carnahan E, Katahoire A. etal.Coverage and Drivers to reaching the last child with vaccination in Urban Settings: a mixed-methods study in Kampala, Uganda.Glob Health Sci Pract.2022;10(4). 10.9745/GHSP-D-21-00663 PMC9426991 36041847

[40] Kajungu D, Hirose A, Rutebemberwa E, Pariyo GW, Peterson S, Guwatudde D (2020). Cohort Profile: the Iganga-Mayuge Health and demographic surveillance site, Uganda (IMHDSS, Uganda). Int J Epidemiol.

[41] Health Care Utilization Theories and Models -Case Western…Internet]. [cited 2021 Apr 20]. Available from: https://www.yumpu.com/en/document/read/18269764/health-care-utilization-theories-and-models-case-western-.

[42] Tolera H, Gebre-Egziabher T, Kloos H (2020). Using Andersen’s behavioral model of health care utilization in a decentralized program to examine the use of antenatal care in rural western Ethiopia. PLoS ONE.

[43] Herliana P, Douiri A (2017). Determinants of immunisation coverage of children aged 12–59 months in Indonesia: a cross-sectional study. BMJ Open.

[44] Immunization Agenda 2030 - A Global Strategy To Leave No One Behind. Available from: https://www.unicef.org/supply/media/10121/file/14-IA2030-Summary-Rajinder-Suri.pdf. 10.1016/j.vaccine.2022.11.042 39004466

[45] Slums: A fierce challenge to Uganda’s urbanisation drive - New Vision Official [Internet]. [cited 2023 Jul 21]. Available from:https://www.newvision.co.ug/news/1304923/slums-fierce-challenge-uganda-urbanisation-drive.

[46] Kwiringira JN, Kabumbuli R, Zakumumpa H, Mugisha J, Akugizibwe M, Ariho P (2021). Re-conceptualizing sustainable urban sanitation in Uganda: why the roots of “Slumification” must be dealt with. BMC Public Health.

[47] Wiysonge CS, Uthman OA, Ndumbe PM, Hussey GD (2012). Individual and contextual factors associated with low childhood immunisation coverage in sub-saharan Africa: a multilevel analysis. PLoS ONE.

[48] Umoke PCI, Umoke M, Nwalieji CA, Igwe FO, Umoke UG, Onwe RN (2021). Investigating factors Associated with Immunization Incompletion of Children under five in Ebonyi State, Southeast Nigeria: implication for policy dialogue. Glob Pediatr Health.

[49] Sarker AR, Akram R, Ali N, Sultana M (2019). Coverage and factors associated with full immunisation among children aged 12–59 months in Bangladesh: insights from the nationwide cross-sectional demographic and health survey. BMJ Open.

