# Supplementary material for: Comparing full immunisation status of children (0–23 months) between slums of Kampala City and the rural setting of Iganga District in Uganda: a cross-sectional study
Source: BMC Health Serv Res. 2023 Aug 14;23:856. doi: 10.1186/s12913-023-09875-w (PMC10424339; doi:10.1186/s12913-023-09875-w)

**S1 Table 1** Ugandan National EPI.

| Vaccine | Recommended schedule with Ugandan EPI | WHO recommended Time range |
| --- | --- | --- |
| BCG + Polio 0 | At birth | At birth - 4 weeks |
| Polio 1, Penta 1, PCV1, Rota | At 6 weeks | 4 weeks - 2 months |
| Polio 2, Penta 2, PCV 2, Rota 2 | At 10 weeks | 8 weeks - 4 months |
| Polio 3, IPV, Penta 3, PCV 3 | At 14 weeks | 12 weeks - 6 months |
| Measles | At 9 months | 9 months - 12 months |

Abbreviations: BCG- Bacillus of Calmette and Guerin; Penta - Pentavalent vaccine (Diphtheria, Pertussis, Tetanus, Haemophilus Influenza B, Hepatitis B); PCV - Pneumococcal Conjugate Vaccine. The national recommended vaccines and the time of administration of the individual dose vaccines. However, these vaccines' boosters and subsequent doses are given up to 18 months.

**S2 Figure 1:** The 2016 UDHS data on childhood immunisation


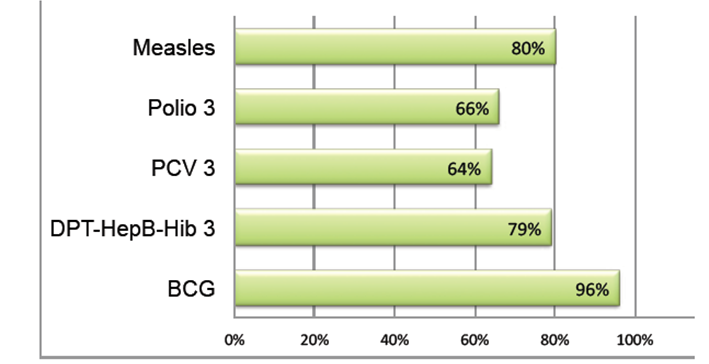


Uganda National immunisation coverage demonstrating a high uptake of BCG vaccine and low coverage of PCV3

**S3 Figure 2:** Percentage of the basic childhood immunisation status of the participants


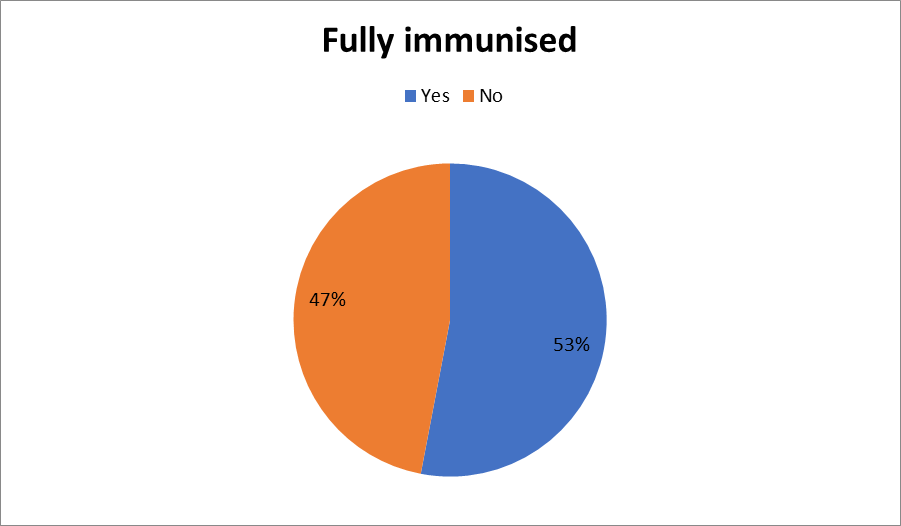

Supplement: Supplementary file 1 — Supplementary Material 1 [file 12913_2023_9875_MOESM1_ESM.docx]
